# Chemotherapy related changes in cfDNA levels in squamous non-small cell lung cancer: correlation with symptom scores and radiological responses

**DOI:** 10.37349/etat.2024.00232

**Published:** 2024-05-28

**Authors:** Nithiyanandan Ravi, Parul Gupta, Amanjit Bal, Kuruswamy Thurai Prasad, Mandeep Garg, Rakesh Kapoor, Navneet Singh

**Affiliations:** Istituto Nazionale Tumori-IRCCS-Fondazione G. Pascale, Italy; Department of Pulmonary Medicine, Postgraduate Institute of Medical Education and Research (PGIMER), Chandigarh 160012, India

**Keywords:** Cell free DNA, squamous, non-small cell lung cancer, chemotherapy, overall survival, progression-free survival

## Abstract

**Aim:**

There is limited data on prognostic value of baseline plasma cell free DNA (cfDNA) in advanced squamous non-small cell lung cancer (sq-NSCLC). This prospective observational study aimed to assess change in plasma cfDNA levels in locally-advanced/metastatic sq-NSCLC with chemotherapy and its correlation with symptom-scores and radiological-responses.

**Methods:**

Chemotherapy-naive patients with stages-IIIB/IIIC/IV sq-NSCLC (*n* = 59), smokers with chronic obstructive pulmonary disease [COPD, COPD-controls (CC); *n* = 27] and healthy-controls (*n* = 25) were enrolled. Respiratory symptom burden (RSB) and total symptom burden (TSB) were calculated from mean visual-analog-scores (VAS) of dyspnoea, cough, chest pain, hemoptysis RSB, anorexia and fatigue (all six for TSB). cfDNA was isolated from peripheral blood. All patients received platinum-doublet chemotherapy. RSB/TSB/cfDNA assessment and contrast-enhanced computed tomography (CECT)-thorax scans were done at baseline and post-chemotherapy.

**Results:**

At baseline, 13/59 (22%) sq-NSCLC, 3/27 (11%) CC and none (0%) healthy-controls had detectable cfDNA. All three CC were heavy smokers with no evidence of malignancy and undetectable cfDNA levels on repeat testing. In sq-NSCLC group, majority were males (95%), current-smokers (88%), heavy-smokers (70%), had metastatic disease (59%) with median age of 65 years. Eastern Co-operative Oncology Group (ECOG) performance status (PS) was 0–1 (56%) and 2 (42%). Median RSB- and TSB-scores were 9 [interquartile range (IQR) = 5–14] and 16 (IQR = 9–23), respectively. Of the 59 patients, 54 received ≥ 1 cycle while 27 underwent post-C4 evaluation with detectable cfDNA levels in 18/27 (66.7%). No baseline characteristic correlated with cfDNA detectability. Median overall survival (OS) and progression-free survival (PFS) were 262 days and 167 days, respectively. ECOG PS ≥ 2, RSB-score > 9 and TSB-score > 16 were all associated with worse OS and PFS as was cfDNA detectability [median OS = 97 days *vs.* 298 days and median PFS = 97 days *vs.* 197 days; *P* = 0.025; hazard ratio (HR) = 2.17].

**Conclusions:**

Baseline cfDNA detectability is independently associated with poor OS and PFS in patients with advanced sq-NSCLC on chemotherapy.

## Introduction

Lung cancer (LC) is the most common cause of cancer related death around the world. Histopathologically, LC can be subdivided into two major subtypes, i.e., non-small cell lung cancer (NSCLC) and small-cell lung cancer (SCLC). NSCLC constitutes approximately 80% of lung cancer cases and the majority of those patients present with locally advanced or metastatic disease at the time of diagnosis. Host factors like performance status (PS), age, weight loss, smoking, symptoms at baseline, staging, hemoglobin, lactate dehydrogenase (LDH) levels, albumin, proteinuria and proliferation markers like Ki-67 and DNA ploidy, etc., are associated with lung cancer prognosis. However, molecular prognostic markers depicting the response and outcome to therapy have still not been identified yet [1, 2]. Traditionally computed tomography (CT, CT scans) is used to assess the response to therapy and relapse. One of the major drawbacks of this approach is delayed detection of relapse/disease progression and a change in therapy at this stage might not be as effective. This delay in detection of response to therapy is owing to the fact that CT scans take into consideration the macroscopic alterations in the tumor rather than the biological activity and heterogenicity of the tumor mass [3]. Recently, molecular biomarkers such as cell free DNA (cfDNA), circulatory tumor cells (CTCs), microRNA (miRNA), exosomes, etc., are being studied for their potential as prognostic biomarkers in the field of cancer biology. Many advances have been achieved in the detection and molecular characterization of CTCs especially amongst patients who have undergone surgical resection, however, still several challenges exist that limit its use as biomarker [4]. Among these, cfDNA has been studied and exploited the most [5].

cfDNA are short double stranded DNA fragments released into the circulation by both tumour and non-tumour tissues. Circulating tumor DNA (ctDNA) is the term used to define the DNA fragments released into the plasma by tumour tissues alone [6]. ctDNA can be differentiated from cfDNA by the detecting the tumor specific alterations [such as single nucleotide variants (SNVs), indels, etc.] in the former [2]. cfDNA are detectable in the plasma of both healthy subjects and those with malignant and non-malignant conditions. However, cfDNA concentrations are higher in persons with malignant or non-malignant disorders than in healthy controls. This is attributed to micrometastases, apoptosis, necrosis, spontaneous release of DNA by tumour cells, angiogenesis, etc. [7]. Non-malignant conditions with higher cfDNA levels in plasma include systemic lupus erythematosus, rheumatoid arthritis, pulmonary embolism, myocardial infarction, trauma and therapeutic procedures. cfDNA concentrations have been found to be higher in the serum or plasma of patients with lung cancer compared to healthy individuals or in those with benign diseases. Squamous NSCLC (sq-NSCLC) is known to harbor higher frequency of mutations and ctDNA levels in the plasma [8].

The current study was designed to assess the prognostic value of cfDNA as a baseline prognostic marker and to correlate the change in plasma cfDNA levels post-chemotherapy with symptom scores and radiological responses (RRs) in locally advanced/metastatic sq-NSCLC.

## Materials and methods

### Study group

This was a prospective observational study conducted at a tertiary care referral centre over an eighteen-month period and enrolled both out-patients and admitted patients.

Inclusion criteria: being a pilot study, 50 consecutive treatment-naive patients with histopathologically and/or cytologically confirmed squamous cell lung cancer, tumor-nodes-metastasis (TNM) 8th edition stages IIIB/IIIC/IV, Eastern Co-operative Oncology Group (ECOG) PS 0–2 and aged 18 years or above were planned for inclusion. Prior radiation treatment was allowed if completed at least 2 weeks prior to chemotherapy initiation.

Exclusion criteria: active infection, patients with chronic inflammatory diseases (like systemic lupus erythematosus and rheumatoid arthritis), trauma requiring surgical intervention (within the past 48 h), acute myocardial infarction (within the past 48 h), pulmonary thromboembolism (within the past 48 h), invasive diagnostic or therapeutic procedures (performed within the past 48 h) were excluded.

Control group: twenty-five patients with chronic obstructive pulmonary disease (COPD, aged 18 years or above) and 25 healthy subjects (aged 18 years or above) were planned to be enrolled from the outpatient department as controls.

For all enrolled patients, baseline demographic characteristics were noted and investigations and symptom score assessments [respiratory symptom burden (RSB) & total symptom burden (TSB)] done as per previously published protocols [9–12]. The study was approved by the institutional ethics committee and informed consent obtained from all patients/participants prior to enrolment.

### cfDNA isolation and analysis

For cfDNA assessment, 5 mL whole blood sample was drawn from all patients/controls enrolled in the study typically within 48 h of acquisition of CT scan. However, in case the CT scan had been obtained earlier as part of diagnostic evaluation, it had to have been within 4 weeks of baseline blood sample. The sample was sent within 2 h in an ethylenediaminetetraacetic acid (EDTA) vial to the lab for processing. cfDNA was isolated using MagMax^®^ cfDNA isolation kit using manufacturer’s protocol. Briefly, plasma isolated from the blood was centrifuged at 16,000 *g* for 10 min to remove all the remaining cells in the sample. Plasma thus obtained was mixed with binding buffer and magnetic beads and vortexed at high speed for 10 min. Samples were then placed on magnetic stand. Following washing with wash buffer and 80% ethanol, cfDNA was eluted in elution buffer. The cfDNA thus obtained was analyzed and quantitated using a chip-based capillary electrophoresis (Agilent, Santa Clara, CA, USA) on Agilent Bioanalyzer 2100^®^ instrument using Agilent DNA High Sensitivity kit as per manufacturer’s guidelines. For quality control purposes concentration of DNA fragments falling in the range of 150–200 bp only was taken as cfDNA and rest were excluded (Figure S1).

### Treatment and follow-up

All enrolled patients with lung cancer received platinum doublet chemotherapy as per standard institutional protocols [13–15]. Repeat contrast enhanced CT scan was done to assess RR using response evaluation criteria in solid tumours (RECIST) 1.1 criteria after completing 4 cycles of chemotherapy (or earlier if indicated). At this time point, repeat cfDNA assessment was also done using the same technique as for baseline (with blood sample being obtained at least 48 h after completion of the 4th cycle of chemotherapy and within 4 weeks of the follow up CT scan). Symptom scores were also reassessed after 4 cycles of chemotherapy. All the patients were followed up after completion of chemotherapy by two monthly outpatient visits. Survival was calculated from date of initiation of chemotherapy till death [overall survival (OS)] and death or disease progression whichever was earlier [progression-free survival (PFS)].

### Statistical analysis

SPSS version 23 software was used to perform the statistical analysis. Continuous variables are expressed as mean with standard deviation (StdDev) and median (inter-quartile range). Categorical variables are expressed as percentages. The baseline cfDNA detectability was correlated with the baseline demographic characteristics, smoking status, ECOG status, symptom score and TNM stage using Spearman’s correlation. On follow up, patients were grouped into those with complete response (CR), partial response (PR), stable disease (SD) and those with progressive disease (PD) using RECIST 1.1 RR criteria. cfDNA detectability after 4 cycles of chemotherapy was correlated with clinical and RR after chemotherapy using Spearman’s correlation. On the basis of the values of the correlation coefficient (*r*), the correlation was considered very weak (0.001 to 0.199), weak (0.200 to 0.399), moderate (0.400 to 0.599), strong (0.600 to 0.799) or very strong (0.800 to 1.000). Survival probability, median PFS and median OS were calculated using the Kaplan-Meier analysis, and group differences were analyzed using the log-rank test. Factors affecting OS were assessed using Cox proportional hazards regression analysis and calculation of hazard ratio (HR) with 95% confidence interval (CI). For all analyses, *P* value < 0.05 was considered as significant.

## Restults

Out of 78 patients, screened from July 2018 till September 2019, 19 patients were excluded (Figure S2) and 59 patients with sq-NSCLC were included in the study population. Fifty-four patients out of 59 received chemotherapy. Mean ± StdDev age of the study population was 64.2 years ± 9.1 years, 56 were males, and 25 had co-morbidities. Majority (88.1%) were current smokers with a mean smoking index of 551.8. Thirty-six (55.0%) had ECOG 0–1 PS. Mean and median RSB scores were 9.8 (StdDev 5.8) and 9.0 [interquartile range (IQR) 5–14] respectively. Mean and median TSB scores were 16.8 (StdDev 9.9) and 16.0 (IQR 9–23), respectively. Thirty-five patients (59.3%) had metastatic disease (Table 1). cfDNA was detectable in 13 (22.0%) patients at baseline with a mean value of 77.3 pg/μL (Figure S3). Amongst the patients with COPD (*n* = 27) and healthy controls (*n* = 25), 3 (11.1%) of the former (mean value of 20.4 pg/μL) and none of the latter had detectable cfDNA levels. On repeat testing, none of the COPD controls showed detectable cfDNA levels (Figure S3).

**Table 1:** Demographic characteristics of patients at baseline and follow up

**Parameters**	**Baseline (*n* = 59), *n* (%)**	**Follow-up (*n* = 27), *n* (%)**
Age in years	64.3 (9.1)^*^	
Male gender	56 (94.9)	
ECOG status		
0–1	33 (55.9)	18 (66.7)
2–4	26 (44.1)	09 (33.3)
KPS		
90–100	20 (33.9)	13 (48.1)
80 and below	39 (66.1)	14 (51.9)
Smoking status		
Never smoker	02 (3.4)	
Current smoker	52 (88.1)	
Former smoker	05 (8.5)	
Smoking index	551.8 (376.4)^*^	
Comorbidities	25 (42.4)	
TNM stage		
IIIB	16 (27.1)	
IIIC	08 (13.6)	
IVA	30 (50.8)	
IVB	05 (8.5)	
RSB score	9.9 (5.8)^*^	4.1 (4.5)^*^
TSB score	16.9 (9.9)^*^	8.2 (8.0)^*^
cfDNA detectability	13 (22.0)	18 (66.7)
cfDNA levels (pg/μL)	77.3 (181.3)^*^	244.3 (279.8)^*^
RECIST 1.1 change		
PR		18 (66.7)
SD		05 (18.5)
PD		4 (14.8)

^*^ Data represented as mean (StdDev). Current smoker: smoked cigarettes/bidis within the last 6 months; never smoker: smoked less than 100 cigarettes in his/her lifetime; former smoker: smoked cigarettes/bidis before 6 months; KPS: Karnofsky performance scale; PR: partial response; SD: stable disease; PD: progressive disease; ECOG: Eastern Co-operative Oncology Group; TNM: tumor-nodes-metastasis; RSB: respiratory symptom burden; TSB: total symptom burden; cfDNA: cell free DNA; RECIST: response evaluation criteria in solid tumours

### Chemotherapy and follow up

Of the 54 patients with sq-NSCLC who received chemotherapy, 48 (88.8%) received taxane-based chemotherapy regimen and 6 (11.1%) received gemcitabine-based chemotherapy. Of these, 44 patients received at least two cycles of chemotherapy, 38 patients received at least three cycles while 10 patients received only one cycle. Fifteen patients received radiotherapy in addition to chemotherapy.

Overall, 27 patients from the study population reached 1st follow-up out of which 18 (66.7%) had detectable cfDNA levels with a mean value of 244.3 pg/μL (StdDev 279.8) and median value of 154 pg/μL (IQR 427). Among these 27 patients, 18 (66.7%) patients had ECOG 0–1 at follow-up. Mean RSB score at follow-up was 4.07 (StdDev 4.5) and mean TSB score was 8.19 (StdDev 8.0). Distribution of RR at follow-up were PR in 18 (66.7%), SD in 5 (18.5%) and PD in 4 (14.8%) patients (Table 1).

Baseline characteristics like gender, ECOG status, smoking status, smoking severity, RSB score, TSB score, Karnofsky performance scale (KPS), TNM 8 stage, presence of comorbidities and hematological parameters had very weak correlation with cfDNA detectability at baseline. Post-chemotherapy (4 cycles) ECOG status, KPS, TSB score, RSB score, weight change, hematological parameters and RR still had very weak correlation with detectability of cfDNA (Table 2).

**Table 2:** Correlation of baseline and post-chemotherapy follow-up parameters with cfDNA detectability

**Baseline and post-chemotherapy correlations**	**Parameters**	**Spearman coefficient**	***P* value**
Correlation of cfDNA detectability at baseline	Gender	–0.12	0.35
	ECOG status	0.19	0.16
	KPS	0.21	0.11
	Smoking status	0.04	0.77
	Smoking severity	–0.09	0.49
	TNM 8 stage	0.18	0.16
	Co-morbidities	0.04	0.76
	RSB score	0.05	0.71
	TSB score	0.05	0.71
	Weight change	–0.08	0.52
Correlation of cfDNA detectability at follow-up (post chemotherapy)	ECOG status	0.00	1.00
	KPS	0.10	0.60
	RSB score	–0.32	0.11
	TSB score	–0.10	0.60
	Weight change	0.10	0.63
	RECIST 1.1 change	–0.02	0.93

cfDNA: cell free DNA; ECOG: Eastern Co-operative Oncology Group; KPS: Karnofsky performance scale; TNM: tumor-nodes-metastasis; RSB: respiratory symptom burden; TSB: total symptom burden

**Figure 1:**
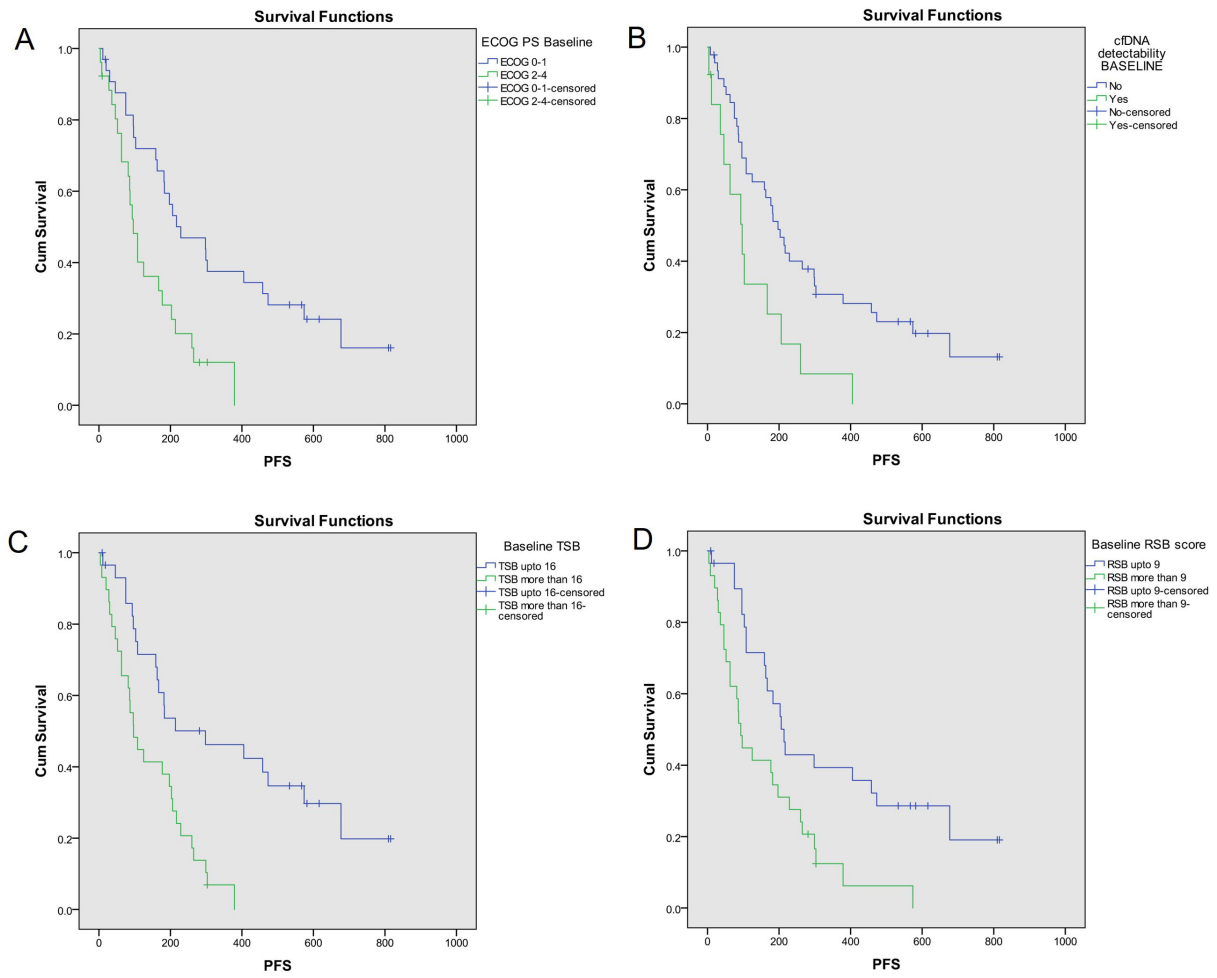
PFS analysis based on key baseline variables [10]. Probability of PFS (Kaplan-Meier analysis) was significantly higher with (A) ECOG PS 0–1 [229 days (95% CI = 89–369)] as compared to ECOG PS 2–4 [96 days (95% CI = 70–122); log-rank *P* = 0.002]; (B) no cfDNA detectability [197 days (95% CI = 148–246)] as compared to cfDNA detectability [97 days (95% CI = 40–154); log-rank *P* = 0.008]; (C) TSB^*^ ≤ 16 [298 days (95% CI = 19–577)] as compared to TSB >16 [97 days (95% CI = 60–134); log-rank *P* < 0.001]; (D) RSB^*^ ≤ 9 [214 days (95% CI = 170–258)] as compared to RSB > 9 [93 days (95% CI = 74–112); log-rank *P* = 0.003]. The asterisk (^*^) indicates that the reference is [10]. CI: confidence interval; PFS: progression-free survival; ECOG: Eastern Co-operative Oncology Group; PS: performance status; cfDNA: cell free DNA; RSB: respiratory symptom burden; TSB: total symptom burden

Median PFS of the study population was 177 days (95% CI = 103–255). Kaplan-Meier analysis (Figure 1) showed median PFS was significantly more with ECOG PS 0–1 [229 days (95% CI = 89–369)] than ECOG PS 2–4 [96 days (95% CI = 70–122); *P* = 0.002], KPS 90–100 [458 days (95% CI = 100–816)] than KPS ≤ 80 [125 days (95% CI = 32–218); *P* < 0.001], no cfDNA detectability [197 days (95% CI = 148–246)] than cfDNA detectability [97 days (95% CI = 40–154); *P* = 0.008], RSB ≤ 9 [214 days (95% CI = 170–258)] than RSB > 9 [93 days (95% CI = 74–112); *P* = 0.003] and with TSB ≤ 16 [298 days (95% CI = 19–577)] than TSB > 16 [97 days (95% CI = 60–134); *P* < 0.001].

**Figure 2:**
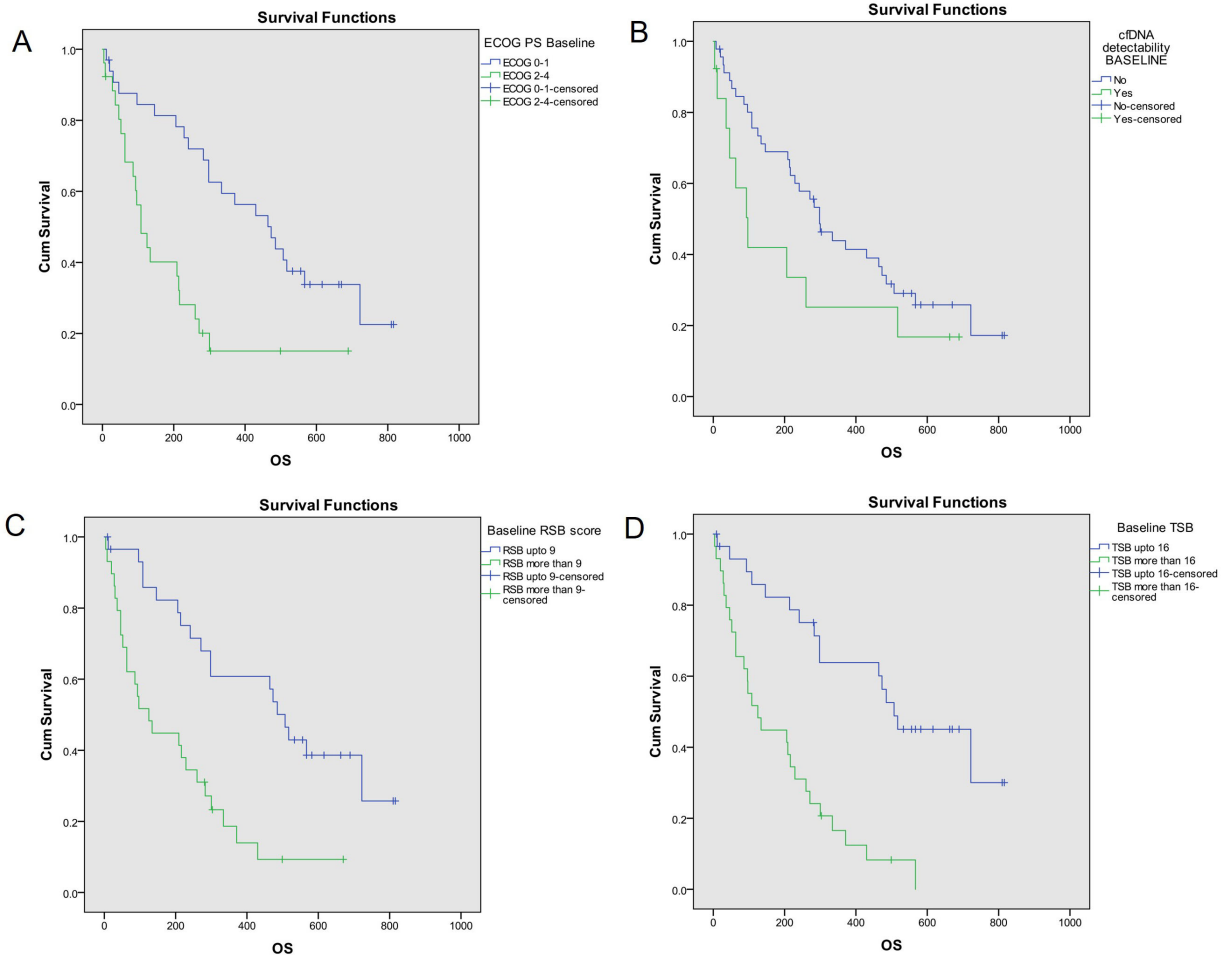
OS analysis based on key baseline variables [10]. Probability of OS (Kaplan- Meier analysis) was higher with (A) ECOG PS 0–1 [473 days (95% CI = 315–631)] as compared to ECOG PS 2–4 [108 days (95% CI = 61–155); log-rank *P* = 0.001]; (B) no cfDNA detectability [298 days (95% CI = 219–377)] as compared to cfDNA detectability [97 days (95% CI = 40–154)]; log-rank *P* = 0.128; (C) TSB^*^ ≤ 16 [507 days (95% CI = 329–685)] as compared to TSB >16 [125 days (95% CI = 60–190); log-rank *P* < 0.001]; (D) RSB^*^ ≤ 9 [507 days (95% CI = 439–575)] as compared to RSB > 9 [125 days (95% CI = 53–197); log-rank *P* < 0.001]. The asterisk (^*^) indicates that the reference is [10]. OS: overall survival; CI: confidence interval; ECOG: Eastern Co-operative Oncology Group; PS: performance status; cfDNA: cell free DNA; RSB: respiratory symptom burden; TSB: total symptom burden

Median OS of the study population was 271 days (95% CI = 186–356). Kaplan-Meier analysis (Figure 2) showed better OS with ECOG PS 0–1 [473 days (95% CI = 315–631)] than ECOG PS 2–4 [108 days (95% CI = 61–155); *P* = 0.001], KPS 90–100 [722 days (95% CI = 422–1022)] than KPS ≤ 80 [206 days (95% CI = 79–333); *P* < 0.001], RSB ≤ 9 [507 days (95% CI = 439–575)] than RSB > 9 [125 days (95% CI = 53–197); *P* < 0.001] and TSB ≤ 16 [507 days (95% CI = 329–685)] than TSB > 16 [125 days (95% CI = 60–190); *P* < 0.001].

Univariate Cox proportional hazard analysis had shown association of low PFS with higher RSB score, higher TSB score, poor ECOG PS, poor KPS and cfDNA detectability at baseline. Multivariate analysis had shown higher TSB score [HR = 2.29 (95% CI = 1.13–4.66), *P* = 0.022] and cfDNA detectability [HR = 2.17 (95% CI = 1.10–4.26), *P* = 0.025] at baseline as independent prognostic markers for low PFS (Table 3). For OS, results were similar as for PFS except that cfDNA detectability did not show an association either on univariate or multivariate analysis.

**Table 3:** Cox proportional hazards regression analysis for factors affecting OS and PFS

**Variable**		**PFS (univariate analysis)**		**PFS (multivariate analysis)**		**OS (univariate analysis)**		**OS (multivariate analysis)**	
		**HR (95% CI)**	***P* value**	**HR (95% CI)**	***P* value**	**HR (95% CI)**	***P* value**	**HR (95% CI)**	***P* value**
Age^*^		0.97 (0.94–1.00)	0.100			0.99 (0.95–1.02)	0.492		
RSB score	≤ 9	1				1			
	> 9	1.06 (1.01–1.12)	0.020			1.09 (1.04–1.15)	0.001		
RSB score^*^		2.39 (1.31–4.35)	0.004			3.28 (1.71–6.32)	< 0.001		
TSB score	≤ 16	1				1			
	> 16	1.03 (1.01–1.07)	0.011	2.29 (1.13–4.66)	0.022	1.05 (1.02–1.08)	0.001	3.64 (1.72–7.71)	0.001
TSB score^*^		2.99 (1.57–5.67)	0.001			4.17 (2.13–8.17)	< 0.001		
ECOG PS	0–1	1				1			
	2–4	2.57 (1.39–4.77)	0.003	1.68 (0.85–3.31)	0.137	2.97 (1.56–5.66)	0.001	1.59 (0.79–3.22)	0.194
KPS	90–100	1				1			
	≤ 80	3.99 (1.87–8.54)	< 0.001			4.28 (1.99–9.23)	< 0.001		
cfDNA detectability	No	1				1			
	Yes	2.40 (1.22–4.70)	0.011	2.17 (1.10–4.26)	0.025	1.73 (0.84–3.52)	0.134	1.78 (0.85–3.72)	0.123
Weight loss	No	1				1			
	Yes	0.50 (0.22–1.13)	0.097			0.46 (0.19–1.10)	0.081		
Smoking index	≤ 300	1				1			
	> 300	1.21 (0.63–2.33)	0.572			1.46 (0.72–2.97)	0.297		
Fatigue	No	1				1			
	Yes	1.57 (0.82–3.02)	0.177			1.73 (0.86–3.47)	0.123		
TNM 8 stage	III	1				1			
	IV	1.50 (0.82–2.74)	0.184			1.48 (0.78–2.80)	0.225		

^*^ Taken as continuous variable. HR: hazards ratio; CI: confidence interval; RSB: respiratory symptom burden; TSB: total symptom burden; OS: overall survival; KPS: Karnofsky performance scale; PFS: progression-free survival; ECOG: Eastern Co-operative Oncology Group; PS: performance status; cfDNA: cell free DNA

## Discussion

In the present study 59 squamous cell carcinoma (sqCC) patients were enrolled to assess the prognostic importance of cfDNA. Out of the 59 patients, 13 had detectable cfDNA levels at baseline giving a prevalence of 22%. cfDNA levels were also found in 3 out of total 27 patients with COPD which on repeat testing were negative for cfDNA levels. Also, none of the healthy controls had detectable cfDNA levels. A study comparing cfDNA levels in patients with various types of cancer *vs.* healthy controls observed the mean values of cfDNA to be significantly higher in cancer patients [16]. A meta-analysis, showed that cfDNA has pooled sensitivity of 80% and specificity of 77% for the diagnosis of lung cancer [17]. Another study showed that patients with NSCLC had significantly higher cfDNA levels as compared to patients with COPD and healthy controls [18]. Various techniques are available to detect or quantitate the cfDNA in blood such as real-time reverse transcription-polymerase chain reaction (RT-PCR), Bioanalyzer^®^, Qubit^®^, quantitative-PCR (qPCR), etc., and hence published studies have used different techniques to analyze cfDNA levels in lung cancer. Kumar et al. [3] used PicoGreen^®^ double-stranded DNA (dsDNA) kit (Molecular Probes, Eugene, OR, USA) to detect cfDNA in 42 NSCLC (including 31 squamous). Another study compared the sensitivity of Bioanalyser^®^ *vs.* RT-PCR in detecting cfDNA and found that both the techniques were equally sensitive [19]. In the current study, results from usage of Bioanalyzer^®^ for measuring the cfDNA levels in blood of patients with lung cancer is consistent with previous studies.

In this study, no correlation was found between any of the baseline characteristics of the study population with cfDNA detectability at baseline. Previous studies have shown inconsistent results as well [7, 20]. cfDNA level does not necessarily correlate with the total tumour burden as observed by positron emission tomography (PET)/CT scan [21]. Yoon et al. [22] studied cfDNA levels using qPCR in patients with lung cancer and healthy individuals and found that although the former had significantly higher cfDNA levels as, smoking status and smoking severity had no correlation with cfDNA levels. Another study also found no association between cfDNA levels and TNM staging in patients with NSCLC [23]. A study by Santos et al. [24] found no association between cfDNA levels and gender, age, ethnicity, PS, stage, histology, or smoking. Also, no association was found between cfDNA levels at baseline and response after two treatment cycles [24]. Therefore, the results from the current study seem to substantiate the hypothesis that baseline characteristics have no consistent relationship with detectability of cfDNA in patients with NSCLC.

cfDNA levels were also determined in 27 patients who received 4 cycles of chemotherapy. The detectability of cfDNA increased from 22% at baseline to 66.7% post chemotherapy. Also, the mean level of cfDNA increased from 77.3 pg/μL to 244.3 pg/μL after post chemotherapy. However, no correlation between cfDNA levels and RR was observed in our study with 12/18 patients with PR, 3/5 with SD and 3/4 with PD having detectable cfDNA levels. Also, post-chemotherapy cfDNA levels had no correlation with symptom scores. It has been postulated that the increase in cfDNA levels during treatment (chemotherapy and radiation) does not necessarily reflect an increase in ctDNA content but possible inflammation and/or tissue necrosis and apoptosis induced by the treatment given leading to release of more DNA in the circulation [25]. Li et al. [5] conducted a prospective study to look for the association between changes in cfDNA levels and radiologic response after systemic therapy in patients with stages IIIB–IV NSCLC. They measured cfDNA levels at baseline and after 6–12 weeks following chemotherapy and observed that baseline and post chemotherapy cfDNA levels had no association with PFS, OS and RR. However, using radiological change as continuous variable, a weak positive correlation was found between change in cfDNA levels and radiologic response [5]. Another study measured cfDNA levels before 1st, 2nd and 3rd cycle chemotherapy in patients with advanced NSCLC and correlated it with RR. A significant difference in levels of cfDNA between those with PD, CR/PR and SD groups was observed. But when the kinetics of cfDNA levels were taken into consideration, there was a significant difference only between SD and CR/PR groups but not between PD and SD groups [3]. On the other hand, Gautschi et al. [1] conducted a study on 91 patients with stages I–IV NSCLC in which CT scans and cfDNA levels were done at baseline and after chemotherapy. The cfDNA concentration post-chemotherapy was significantly higher than at baseline in the PD group, and not in the PR/SD group. Other studies have reported that cfDNA levels decreased post-treatment in PR but not in those with PD and SD [26] and an increase in cfDNA levels in SD/PD group but not in the PR group [27]. Davis et al. [28] observed that cfDNA can be used to monitor progression in patients with advanced NSCLC before clinical or radiological progression. However, the limited evidence available has not led to cfDNA detectability being recommended for use as a prognostic biomarker for patients undergoing systemic therapy in routine clinical practice.

In the current study, high symptom scores and detectable cfDNA levels were observed to be associated with worse survival (both OS and PFS) something that is consistent with our previous observations/studies utilising graded symptom score assessment [11]. In addition, baseline cfDNA detectability was also associated with worse PFS on multivariate analysis. This is consistent with previous studies (Table 4). Baseline cfDNA levels may identify patients at high risk for poor survival [29]. In addition, patients with higher than median baseline cfDNA levels had been observed to have a significantly worse OS than those less than median. In another study, it was found that higher baseline cfDNA were associated with poor OS (10 months) than those with lower concentrations (14.2 months). However, this study did not establish any relationship between baseline cfDNA levels and the response to chemotherapy—something that was also noted in the current study [30]. In a large prospective trial, it was found that high levels of baseline cfDNA had significantly negative prognostic value on OS and PFS but the percentage change in cfDNA following treatment did not differ significantly between the various treatment response groups as assessed by CT scans [20]. A meta-analysis highlighted prognostic significance of baseline high cfDNA concentrations in patients with lung cancer and found poor OS in those individuals [31]. Soliman et al. [18] showed that lower cfDNA levels at baseline were associated with better OS. Pontes et al. [23] found that detectability of cfDNA at baseline in patients with NSCLC has poor prognostic value.

**Table 4:** Summary of studies involving NSCLC patients in whom cfDNA testing was done

**Authors**	***N***	**Stage/Histology**	**Treatment**	**cfDNA baseline**	**cfDNA post treatment**	**Survival reported**	**cfDNA association**
Gautschi et al. [1]	185	All (34% stage III; 56% stage IV), all NSCLC (26% sqCC)	Chemotherapy (*n* = 127; 69%)	Yes	Yes	Median OS = 7.1 month	A/w poor OS (inconsistent C/w RR)
Kumar et al. [3]	42	III–IV (45% stage IV), all NSCLC (74% sqCC)	Chemotherapy	Yes	Yes	Median OS = 12.0 month (for patients receiving ≥ 3 cycles)	Inconsistent A/w OS (C/w RR)
Li et al. [5]	103	IIIB–IV, all NSCLC (16% sqCC)	Chemotherapy and/or tyrosine kinase inhibitors (TKI, *n* = 86; 83%)	Yes	Yes	OS and PFS (median values not reported)	No A/w PFS & OS (also no C/w RR)
Soliman et al. [18]	60	All (31% stage III; 53% stage IV), all NSCLC (< 33% sqCC)	Observation (chemotherapy in 2; 3%)	Yes	No	OS (median values not reported)	A/w poor OS
Hyun et al. [20]	112	All stage IV, all adenocarcinoma	Chemotherapy	Yes	Yes	Median OS = 22.2 month and median PFS = 7.6 month	A/w poor PFS & OS (no C/w RR)
Nygaard et al. [21]	53	III–IV (68% stage IV), all NSCLC (36% sqCC)	Chemotherapy	Yes	No	Median OS = 15.7 month and median PFS = 5.9 month	A/w poor OS but not PFS
Pontes et al. [23]	38	All (26% stage III; 37% stage IV), all NSCLC (53% sqCC)	Not defined	Yes	No	OS (median values not reported)	No A/w OS
Santos et al. [24]	31	III–IV, all NSCLC (39% sqCC)	Chemotherapy	Yes	Yes	Not defined	No C/w RR
Coco et al. [29]	73	All stage IV, all NSCLC (19% sqCC)	Chemotherapy	Yes	No	Median OS = 8.0 month and median PFS = 4.7 month	A/w poor OS (inconsistent C/w RR)
Tissot et al. [30]	218	IIIB/IV (81% stage IV), all NSCLC (20% sqCC)	Chemotherapy	Yes	Yes	Median OS = 12.3 month and median PFS = 6.9 month	A/w poor PFS & OS (no C/w RR)
Current study	59	IIIB–C/IV (59% stage IV), all sqCC	Chemotherapy (*n* = 52; 92%)	Yes	Yes	Median OS = 9.0 month and median PFS = 5.9 month	A/w poor PFS & OS (no C/w RR)

NSCLC: non-small cell lung cancer; cfDNA: cell free DNA; sqCC: squamous cell carcinoma; OS: overall survival; RR: radiological response; PFS: progression-free survival; C/w: correlation with; A/w: association with; *n*: patient number

The major limitation of our study was the small number of patients (being a pilot). Hence the inability to demonstrate an association of baseline demographic characteristics with cfDNA detectability as well as lack of difference in cfDNA detectability between patients with different categories of RR could be a result of the small patient numbers. Another limitation is that the study was limited to patients with squamous histology and therefore its results cannot be extrapolated to other histological types of NSCLC and SCLC patients or to patients being treated with targeted therapies and immunotherapy/chemo-immunotherapy combinations. The latter are now the standard of care for oncogene driven NSCLC and non-oncogene driven NSCLC respectively. However, the limitations notwithstanding, the current study adds to the small body of evidence regarding this potential prognostic biomarker in resource constrained settings wherein access to novel immunotherapeutic drugs is limited or is unaffordable to a vast majority of eligible patients [32].

In summary, baseline cfDNA detectability was observed in almost a quarter of patients with advanced/metastatic squamous NSCLC in this prospective cohort study and was not seen in healthy controls or non-lung cancer COPD patients. Baseline cfDNA detectability did not correlate with any of the baseline demographic characteristics or with any pre-treatment variables. Similarly, no correlation was found between plasma cfDNA detectability and clinical or RR after chemotherapy. Baseline cfDNA was independently associated with poor survival amongst patients with squamous NSCLC receiving platinum doublet chemotherapy.

## Abbreviations

CC: chronic obstructive pulmonary disease-controls
cfDNA: cell free DNA
CI: confidence interval
COPD: chronic obstructive pulmonary disease
CR: complete response
CT: computed tomography
ctDNA: circulating tumor DNA
ECOG: Eastern Co-operative Oncology Group
HR: hazard ratio
IQR: interquartile range
KPS: Karnofsky performance scale
OS: overall survival
PD: progressive disease
PFS: progression-free survival
PR: partial response
PS: performance status
RECIST: response evaluation criteria in solid tumours
RRs: radiological responses
RSB: respiratory symptom burden
SD: stable disease
sqCC: squamous cell carcinoma
sq-NSCLC: squamous non-small cell lung cancer
StdDev: standard deviation
TNM: tumor-nodes-metastasis
TSB: total symptom burden

## Supplementary materials

The supplementary material for this article is available at: https://www.explorationpub.com/uploads/Article/file/1002232_sup_1.pdf.

## Declarations

### Availability of data and materials

The datasets for this manuscript are not publicly available because of institutional and national regulatory guidelines. However, reasonable requests for accessing the datasets should be directed to the corresponding author.

### Copyright

© The Author(s) 2024.
