# Supplementary material for: Chemotherapy related changes in cfDNA levels in squamous non-small cell lung cancer: correlation with symptom scores and radiological responses
Source: Explor Target Antitumor Ther. 2024 May 28;5(3):508–21. doi: 10.37349/etat.2024.00232 (PMC11222716; doi:10.37349/etat.2024.00232)
Supplement: Supplementary file 1 [file 1002232_sup_1.pdf]

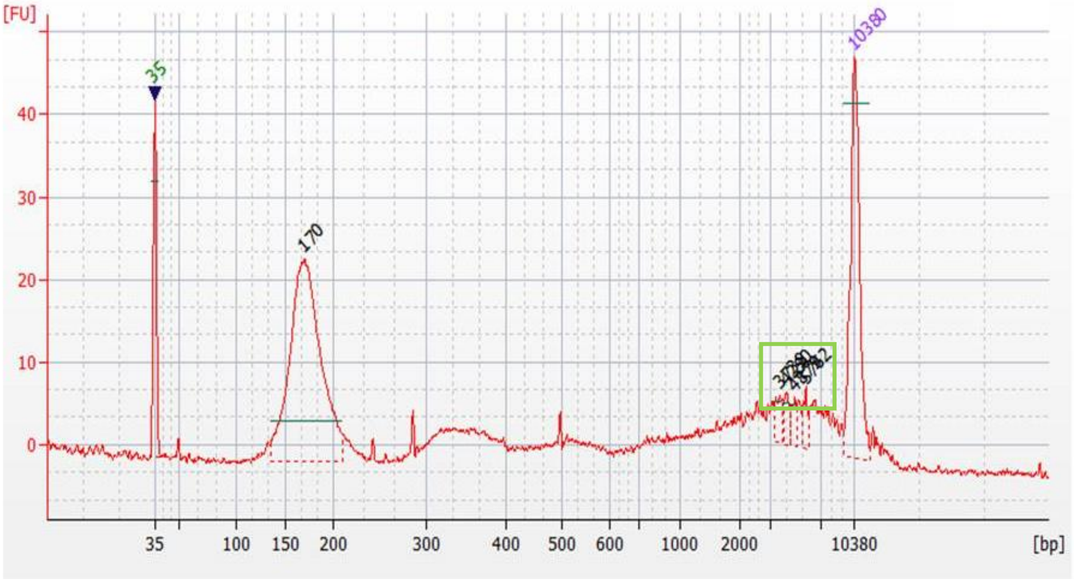

Figure S1. Representative figure of bioanalyzer data from one of the patient showing a peak at 170bp. DNA concentration falling within the range of 150bp-200bp was taken as true cell-free DNA and rest of the peaks were excluded

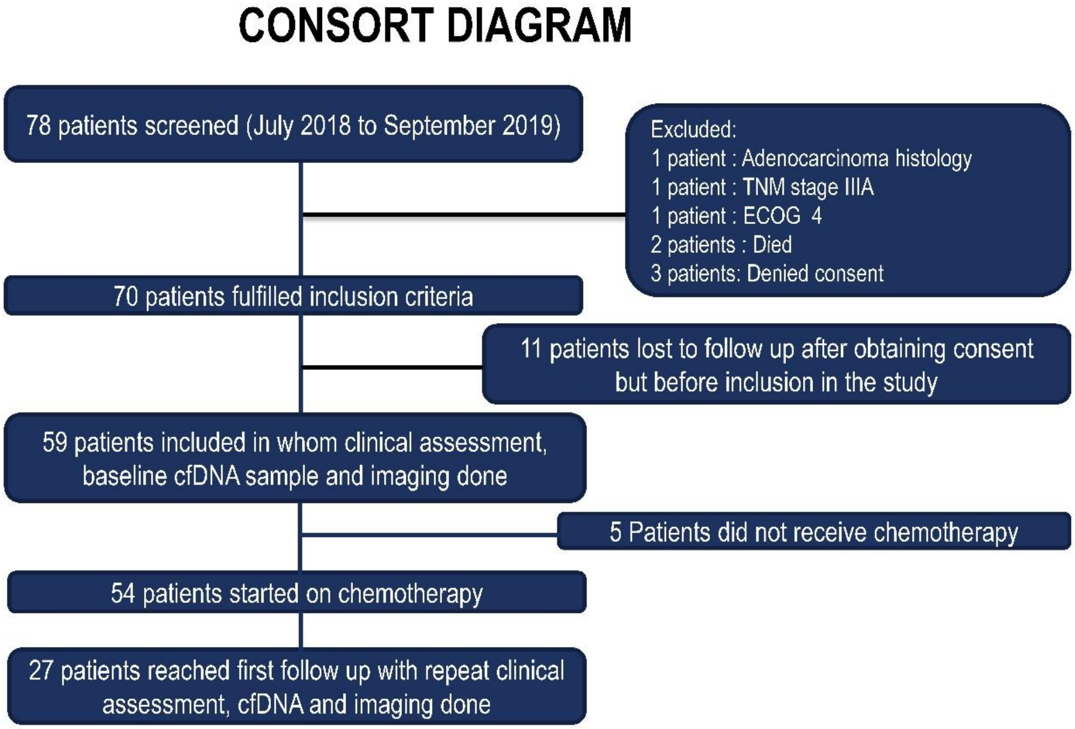

Figure S2. Consort diagram representing the flow of patients in the study

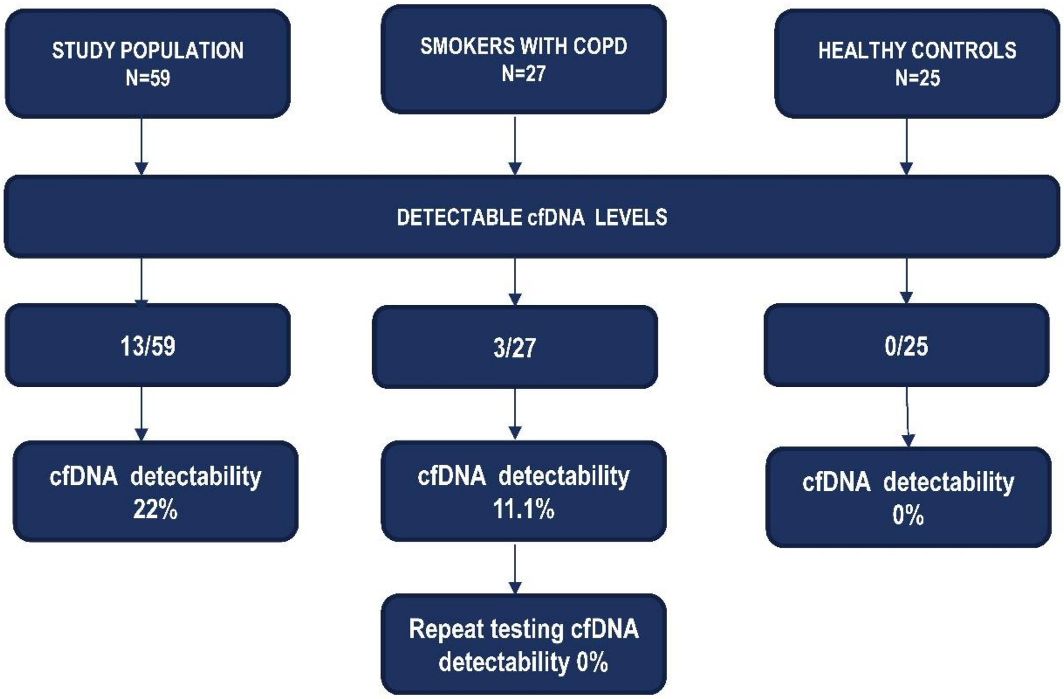

Figure S3. Consort diagram explaining the detectability of cfDNA in blood of patients with lung cancer versus controls having COPD and healthy controls
